# Identification of Growth-Promoting Bacterial Resources by Investigating the Microbial Community Composition of *Polyporus umbellatus* Sclerotia

**DOI:** 10.3390/jof10060386

**Published:** 2024-05-27

**Authors:** Tianrui Liu, Rui Cheng, Zhongyi Hua, Haiyun Gao, Chu Wang, Hui Li, Yuan Yuan

**Affiliations:** 1 Institute of Traditional Chinese Medicine Health Industry, China Academy of Chinese Medical Sciences, Nanchang 330115, China; raymond50@live.cn (T.L.); haiyungao2023@163.com (H.G.); wchu1011@163.com (C.W.); lihuiyiren@163.com (H.L.); 2 Jiangxi Health Industry Institute of Traditional Chinese Medicine, Nanchang 330115, China; 3 State Key Laboratory for Quality Ensurance and Sustainable Use of Dao-di Herbs, National Resource Center for Chinese Meteria Medica, China Academy of Chinese Medical Sciences, Beijing 100700, China; 15922378185@163.com (R.C.); njbxhzy@hotmail.com (Z.H.); 4 Experimental Research Center, China Academy of Chinese Medical Sciences, Beijing 100700, China

**Keywords:** high-throughput sequencing, sustainable cultivation, *Polyporus umbellatus* polysaccharide, combined bacterial liquid, bioactive microbial resources

## Abstract

The sclerotium of the edible mushroom *Polyporus umbellatus* (Zhuling) exhibits various medicinal properties. However, given its long growth cycle and overexploitation, wild resources are facing depletion. Macrofungal growth depends on diverse microbial communities; however, the impact of soil bacteria on *P. umbellatus* development is unknown. Here, we combined high-throughput sequencing and pure culturing to characterize the diversity and potential function of bacteria and fungi inhabiting the *P. umbellatus* sclerotium and tested the bioactivities of their isolates. Fungal operational taxonomic units (OTUs) were clustered and classified, revealing 1275 genera. Bacterial OTUs yielded 891 genera. Additionally, 81 bacterial and 15 fungal strains were isolated from *P. umbellatus* sclerotia. Antagonism assays revealed three bacterial strains (FN2, FL19, and CL15) promoting mycelial growth by producing indole-3-acetic acid, solubilizing phosphate, and producing siderophores, suggesting their role in regulating growth, development, and production of active compounds in *P. umbellatus*. FN2-CL15 combined with bacterial liquid promoted growth and increased the polysaccharide content of *P. umbellatus* mycelia. This study reports new bioactive microbial resources for fertilizers or pesticides to enhance the growth and polysaccharide accumulation of *P. umbellatus* mycelia and offers guidance for exploring the correlation between medicinal macrofungi and associated microbial communities.

## 1. Introduction

*Polyporus umbellatus*, also known as Zhuling, is a renowned edible and medicinal mushroom that is widely distributed in China, Korea, Japan, Southeast Asia, Europe, and North America. For the past 2500 years, the dried sclerotium of *P. umbellatus* has been utilized in traditional Chinese medicine for addressing edema and harnessing its diuretic properties [1]. Recently, *P. umbellatus* polysaccharides (PUPs) have been demonstrated to possess anti-tumor, liver-protective, and immunomodulatory properties [2]. Medicinal PUP sources are being extensively harvested; however, increasing commercial demand coupled with inadequate protection measures have resulted in overharvesting, significantly depleting wild *P. umbellatus* resources in China [3]. The growth and development of *P. umbellatus* encompass several stages, including mycelium formation, sclerotium development, fruiting body maturation, and spore production. Currently, the artificial cultivation of *P. umbellatus* mainly relies on sclerotium seedlings, which provide nutrients for their growth through symbiosis with *Armillaria* species. Immature sclerotium requires 3–5 years to mature, resulting in a long growth cycle of artificial cultivation. Therefore, it is essential to devise novel cultivation techniques aimed at reducing the production cycle of *P. umbellatus*, thus enhancing its economic viability.

Soil bacteria and fungi play a significant role in shaping the growth and development of numerous commercial mushroom species [4]. In *Pleurotus* and *Agaricus*, the growth of mycelium and the subsequent production of commercially viable fruiting bodies are dependent not only on the intrinsic characteristics of the mycelium but also on the presence and interactions of bacteria and fungi within the substrate. However, the effects of fungi, other than *Armillaria*, and soil bacteria on the growth of *P. umbellatus* remain unclear. Bacteria play critical roles in mushroom growth, including interactions with fungal mycelia and induction of fruiting body formation. In a prior investigation, 23 isolates of *Agaricus bisporus* were identified as effective mushroom growth-promoting bacteria (MGPB). Notably, *Pseudomonas putida* strains Bt4 and Ps7 caused the most significant enhancement in *A. bisporus* production [4]. MGPB can promote mushroom growth through a variety of mechanisms. For example, *Micromonospora lupini* promotes mycelial growth of *Pleurotus ostreatus* by reducing host spawning run time [5], and the *Rahnella* strain HPDA25 secretes indole-3-acetic acid (IAA) to enhance the brunches and growth rate of *A. gallica* rhizomorphs [6]. Although *P. umbellatus* sclerotium can acquire nutrients directly from *Armillaria* spp., it can also absorb phosphorus from the soil. In addition, some MGPB strains solubilize organic and inorganic phosphorus and produce glycosides to promote mushroom growth. Notably, both wild and cultured macrofungal sclerotia grow and develop in symbiosis with diverse microbial communities. Nevertheless, the presence, bioactivity, and functions of bacteria and fungi associated with the *P. umbellatus* sclerotium remain unexplored.

In the present study, we employed high-throughput sequencing and co-culture techniques to systematically survey the bacterial and fungal community structure of the *P. umbellatus* sclerotium and identify MGPB for inoculation. To the best of our knowledge, this study represents the first endeavor to explore the diversity of bacteria and fungi linked to the *P. umbellatus* sclerotium to identify and extract active bacterial and fungal companions capable of modulating the growth, development, and biosynthesis of valuable metabolites in the *P. umbellatus* sclerotium.

## 2. Materials and Methods

### 2.1. P. umbellatus Cultivation and Harvest

In 2018, *P. umbellatus* sclerotia were cultivated in woodland or farmland habitats in Ningshan, Shaanxi Province, China (33°309″ N, 108°828″ E). Chestnut trees grow in the woodland, with abundant fallen leaves. The farmland is adjacent to the chestnut grove; the plot is not shaded by trees and has less leaf fall. Twelve plant holes, each measuring 60 cm in length, 60 cm in breadth, and 10 cm in depth, were excavated in each habitat. Five *A. gallica*-infected wooden sticks (10 cm in diameter, 30 cm in length) were planted in each plant hole together with 2 kg of immature *P. umbellatus* sclerotia. Mature *P. umbellatus* sclerotia were harvested 2 years after planting, and *P. umbellatus* sclerotium samples were collected from the two habitats: *P. umbellatus* sclerotia grown in the woodland (WB) habitat and *P. umbellatus* sclerotia grown in the farmland (FB) habitat. Fresh weight was measured to determine the biological yield. Mature *P. umbellatus* sclerotia from each plant hole were removed from the surface soil and weighed as the fresh weight. The mature *P. umbellatus* sclerotia were then freeze-dried and ground into a fine powder.

### 2.2. Collection of Soil Samples

Soil samples were collected using the five-point sampling method [7]. All soil subsamples from the same plot and sampling period were homogenized and combined to form a composite sample, which was transported to the laboratory in a cooler with dry ice. It was then stored in a refrigerator at −80 °C for DNA extraction and high-throughput sequencing. The control sites, designated UWB and UFB, represented bulk soils from woodland and farmland, respectively. WBP denotes soil from *P. umbellatus* sclerotia cultivated in woodlands, and FBP denotes soil from *P. umbellatus* sclerotia cultivated on farmland.

### 2.3. Ergosterol Content Determination

A total of 0.5 g *P. umbellatus* sclerotia powder was mixed with 10 mL of methanol extracted for 1 h in an ultrasonic bath at 220 W and 50 KHz. The ergosterol was obtained via centrifugation (4 °C) for 10 min at 6797× *g*. The ergosterol content was determined using a UPLC system (Waters, Milford, MA, USA). Ergosterol content was measured as previously described [8]. Six biological replicates were used for each experiment.

### 2.4. Polysaccharides Content Determination

A total of 0.5 g powder of *P. umbellatus* samples underwent extraction using water at 90 °C (10 mL) for 30 min in an ultrasonic bath operating at 250 W and 40 KHz. The resulting supernatant was then mixed with 95% (*v*/*v*) ethanol to achieve a final concentration of 80% (*v*/*v*) ethanol and incubated overnight at 4 °C. Subsequently, PUPs were isolated via centrifugation (4 °C) at 6797× *g* for 10 min. PUPs were then dissolved in 25 mL of water and quantified using the anthrone–sulfuric acid method [9]. Six biological replicates were used for each experiment.

### 2.5. DNA Extraction and 16S rRNA/ITS Gene Sequencing

The cetyltrimethylammonium bromide (CTAB)-based approach was applied to extract soil genomic DNA. Four soil samples were sampled from each habitat. The primers 515F and 806R were used for bacterial 16S rRNA genes [10]. The primers ITS5-1737F and ITS2-2043R were used to target the fungal ITS1 region [11]. The PCR system and conditions were consistent with prior work [6]. PCR product quantification, purification, library preparation, and 16S rRNA/ITS amplicon sequencing were performed by Novogene Corporation (Beijing, China). The Illumina HiSeq 2500 Sequencing Platform enabled 16S rRNA/ITS amplicon sequencing, and 250 bp paired-end reads were generated.

### 2.6. Diversity of Microbial Communities Associated with P. umbellatus

The obtained data with Illumina HiSeq amplicon sequences were processed using the QIIME 2 (Quantitative Insights into Microbial Ecology 2) software platform. The operational taxonomic units (OTUs) were identified as bacteria in the 16S OTU table and as fungal OTUs in the ITS OTU table. Species annotation analysis of the OTU sequences was conducted using the QIIME 2 software and the SSU rRNA database SILVA132. Additional analyses of bacterial and fungal community diversity were carried out using NovoMagic v3.0, accessible at https://magic.novogene.com (accessed on 12 May 2022). The microbial co-occurrence interaction network was explored using R (version 3.3.0), and the network correlation diagram was generated using Gephi software version 0.9.2 [12]. TBtools-II (version 2.019) was utilized for sequence alignment [13].

### 2.7. Isolation and Identification of Microbial Communities Associated with P. umbellatus

A total of 10 g soil on the surface of *P. umbellatus* sclerotia was added in 50 mL of PBS buffer with ultrasound (160 W, 40 kHz, and 30 min). The solutions were prepared by diluting 10^4^-fold. Subsequently, 200 µL of the diluted solution was spread onto various bacterial media, including nutrient agar, Luria–Bertani medium (LB), yeast mannitol agar, Reasoner’s 2A agar, and tryptone soya agar. For fungal isolation, 200 µL of the diluted solution was evenly spread over potato dextrose agar (PDA) medium supplemented with 0.05 g/L streptomycin sulfate. Plates were maintained at 25 °C for 2–5 days [14].

PCR was used to amplify the *16S* rRNA gene with primers 27F and 1492R [15] and the ITS region with primers ITS1 and ITS4 [16]. The PCR system and conditions were in accordance with prior work [6]. PCR products were sent to BGI Genomics (Beijing) Co., Ltd. (Beijing, China). for sequencing. EzBioCloud and NCBI BLAST were used for sequence identification [17,18].

### 2.8. Co-Culture Experiments with P. umbellatus Mycelium

The impact of bacteria and a mixture of bacterial liquids on the growth of *P. umbellatus* was assessed. The bacteria or the combined bacterial liquid were cultured overnight in LB medium at 25 °C and 200 rpm until reaching an optical density (OD_600_) of 0.5. Disks measuring 6 mm in diameter, derived from *P. umbellatus* mycelium, were placed at the center of plates containing a modified composition consisting of 20 g/L glucose, 5 g/L yeast extract fermentation, 1 g/L KH_2_PO_4_, 1 g/L MgSO_4_, and 15 g/L agar. The bacteria or combined bacterial liquid were spread at a 2 cm distance from the disk. *P. umbellatus* and fungal disks, each with a diameter of 6 mm, were positioned on a PDA plate, spaced 2 cm away from the center. Control plates lacking bacteria, fungi, or a combination of both were included. All plates were incubated in darkness at a temperature of 2 5 °C for 20 days. The experiment was conducted with six replicates (*n* = 6). The diameter of the *P. umbellatus* mycelium was measured.

### 2.9. Production of IAA

IAA generation was identified via the Salkowski colorimetric test solution, prepared by dissolving 12 g of FeCl_3_ in 300 mL of ddH_2_O and then adding 429.7 mL of 98% H_2_SO_4_ slowly. The solution was cooled to a set volume of 1 L. The capacity of the three isolates to secrete IAA was assessed using the Salkowski colorimetric technique [19]. IAA production assays were conducted in triplicate, with a minimum of three independent experiments performed.

### 2.10. Phosphate Solubilization

The bacterial culture was grown on Pikovskaya’s agar medium and maintained at a temperature of 28 °C for 5 days. During this time, the plates were observed for the formation of a clear halo around the bacterial colonies, indicating phosphate solubilization activity [20]. The transparent halo and colony diameters were measured to calculate the ratio of the transparent halo to colony diameter (D/d value).

### 2.11. Siderophore Production

A log-phase bacterial culture was inoculated onto chrome azurol sulfonate (CAS) agar plates and incubated at 30 °C for 48 s. Following incubation, plates containing the inoculated colonies were inspected for the appearance of an orange halo surrounding the colonies on the blue agar medium [21]. The orange halo and colony diameters were measured to calculate the ratio of the orange halo to the colony diameter (D/d value).

### 2.12. Nitrogen Fixation

The isolates were introduced into malate Ashby’s nitrogen-free solid medium vials and incubated at 28 °C for 4–7 days to observe their growth conditions [22]. Each experiment was conducted three times.

### 2.13. Antagonism Assays

The isolates FN2, FL19, and CL15 were inoculated into the LB medium in 50-mL centrifuge tubes, followed by a 24 h shaking incubation at 200 rpm and 25 °C to assess their antagonistic activity. The bacterial suspension was adjusted to an ideal absorbance of OD_600_ = 0.5. The LB culture medium plate was equally divided into three areas. Subsequently, 20 µL of 3 bacterial suspensions were spread on each area and left to incubate at 25 °C for 3 days. Strain antagonism was observed at the junction of different regions [23].

### 2.14. Bacterial Consortium Inoculants

A bacterial solution containing FN2, FL19, and CL15 was combined in equal proportions and cultivated in an LB liquid medium. The mixture was then subjected to a 24 h shaking incubation at 200 rpm at 25 °C [24].

### 2.15. Statistical Analysis

Analyzed microbial communities using the R statistical program, version 3.3.0. Student’s *t*-test was used to assess the differences between the control and treatment groups. Data are displayed as means ± SEM; *, *p* < 0.05; **, *p* < 0.01.

## 3. Results

### 3.1. Yield, Ergosterol, and Polysaccharides Content in P. umbellatus

**Figure 1:**
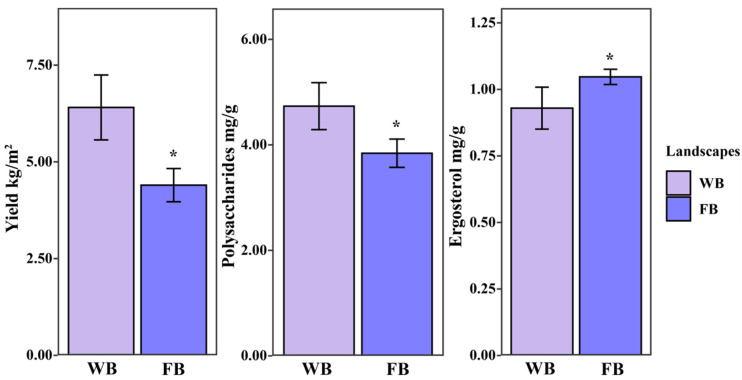
Yield, ergosterol, and polysaccharide contents of *Polyporus umbellatus* sclerotia. WB: *P. umbellatus* sclerotia cultivated in woodland. FB: *P. umbellatus* sclerotia cultivated in farmland. PUPs: *Polyporus umbellatus* polysaccharides. The data are presented as means ± SEM. *, *p* < 0.05.

After 2 years of cultivation, *P. umbellatus* sclerotia established a symbiosis with *A. gallica*, and different habitats affected its yield, ergosterol, and PUP contents. The yield of WB (*P. umbellatus* sclerotia grown in woodland) was 6.40 kg/m^2^, which was 1.45-fold higher than that of FB (*P. umbellatus* sclerotia grown in farmland). Furthermore, the content of PUPs in WB (4.73 mg/g) exceeded that in FB (3.84 mg/g), whereas the ergosterol content in WB (0.93 mg/g) was significantly lower (*p* < 0.05) than that in FB (1.05 mg/g) (Figure 1).

Given the influence of soil microhabitats on soil nutrients, which are significantly affected by soil microbial communities, these results suggest that the composition of soil microbial communities in the two cultivated habitats may impact the yield and the ergosterol and PUP contents of *P. umbellatus*.

### 3.2. Microbial Communities in the Soil of the P. umbellatus Habitat

**Figure 2:**
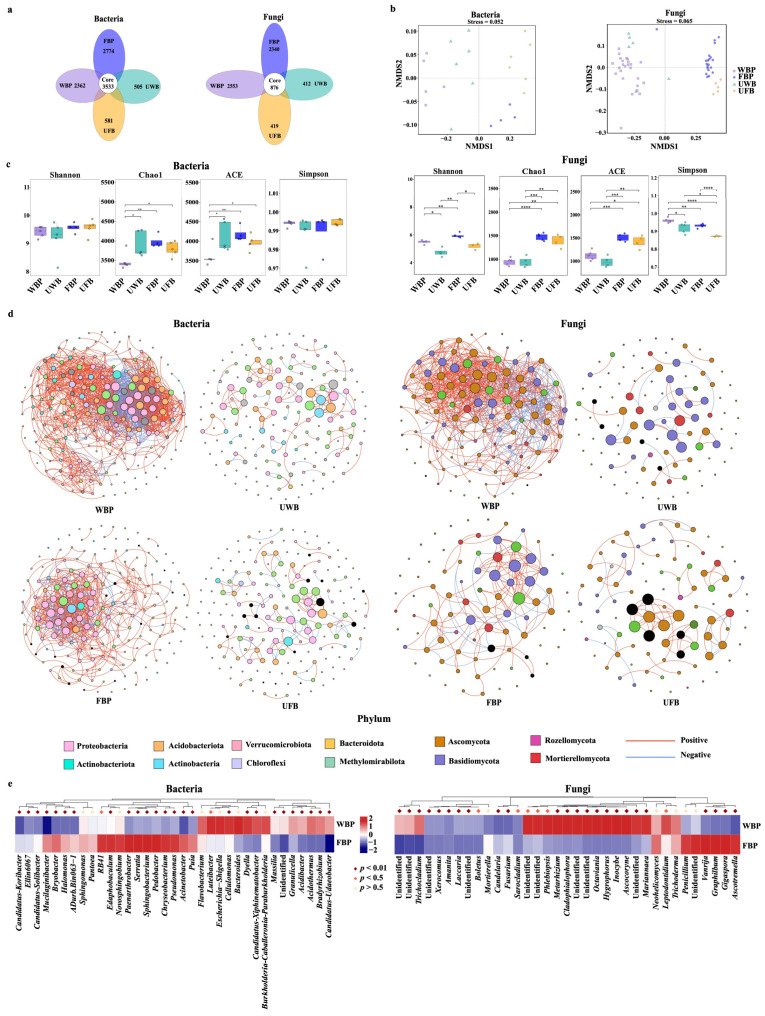
Microbial communities in the soil of the *Polyporus umbellatus* habitats. (**a**) Venn map of microbial communities. (**b**) Non-metric multidimensional scaling analysis of microbial beta diversity. (**c**) Alpha diversity of microbial communities. (**d**) Co-occurring interactions in microbial communities. (**e**) Significantly different microbial communities in different groups as obtained using MetaStat analysis. WBP, soil from *P. umbellatus* sclerotia in the woodland habitat; UWB, bulk soil in the woodland habitat; FBP, soil from *P. umbellatus* sclerotia in the farmland habitat; UFB, bulk soil in farmland habitat. *, *p* < 0.05; **, *p* < 0.01; ***, *p* < 0.001; ****, *p* < 0.0001.

We obtained a total of 19,993 OTUs from the soil samples. The core OTU number of the four soil samples was 3533, accounting for 17.7% of the total OTUs. We obtained 2362, 2774, 505, and 581 bacterial OTUs from WBP, FBP, UWB, and UFB samples, respectively. We also identified 876 fungal OTUs, accounting for 13.2% of the total OTUs. This result indicates that the microbial composition of *P. umbellatus* habitat sites (WBP or FBP) differed from that of the bulk soil sites (UWB or UFB). The number of OTUs in WBP and FBP was higher than that in UWB and UFB (Figure 2a).

We further conducted a non-metric multidimensional scaling (NMDS) analysis using the binary-card distance to reveal consistent differences in β-diversity. The stress values for fungal and bacterial NMDS were 0.065 and 0.052, respectively. All NMDS stress values were less than 0.1 (Figure 2b). The separation of both bacterial and fungal communities from the four soil samples confirmed that habitat conditions could influence microbial communities during the growth of *P. umbellatus*.

Figure 2c shows the alpha indices of the four soil samples, including the richness estimator (Chao1 and ACE) and the diversity indices (Shannon and Simpson). The lowest Chao1 and ACE index of soil bacteria were observed for WBP, which were significantly different from those for UWB (*p* < 0.05), FBP (*p* < 0.01), and UBP (*p* < 0.05). The Simpson’s index of WBP was higher than that of UWB; however, the difference was not statistically significant (*p* > 0.05). These results confirm that bacterial richness was ultimately reduced in WBP. Moreover, the habitat types substantially impacted the composition of fungal communities, as demonstrated by the Chao1, ACE, and Simpson indices, and FBP exhibited a more diverse fungal community than WBP.

The co-occurring microbial interaction network suggested that the cultivation of *P. umbellatus* led to more frequent bacterial or fungal interactions. In co-occurring bacterial interactions, the phyla Proteobacteria, Acidobacteria, and Actinobacteria frequently interacted. Moreover, the relative abundance of Proteobacteria was higher in WBP than in FBP, UWB, and UFB (Table S1), indicating that the abundance and diversity of Proteobacteria may be crucial in the growth of *P. umbellatus*. At the genus level, the significantly different bacterial communities between WBP and FBP revealed that 11 genera were more abundant in WBP, whereas 17 genera were more abundant in FBP (Figure 2e). Moreover, in FBP, the relative abundance of *Pseudomonas* and *Acinetobacter* was 3.08- and 54.51-fold higher than in WBP, respectively, suggesting that these two genera may play a role in promoting the growth of *P. umbellatus* (Table S2).

In the fungal communities, Ascomycota and Basidiomycota were the key phyla in the co-occurring interaction network (Figure 2d). Analysis of the relative abundances also confirmed that the dominant phyla were Ascomycota and Basidiomycota in *P. umbellatus* cultivation. The relative abundance of Ascomycota was 53.24% and 23.57% for WBP and UWB, respectively. Nonetheless, the relative abundance of Ascomycota was reduced in FBP to 41.95% compared to 50.11% in UFB. For the Basidiomycota phylum, after *P. umbellatus* cultivation, the relative abundance decreased in WBP but increased in FBP compared to the bulk soils (Table S3). At the genus level, the relative abundance of *Fusarium* was 3.69-fold in FBP compared with that in WFB (Table S4). These results indicate that the decrease in yield may be related to an increase in Basidiomycota and *Fusarium* abundance.

### 3.3. Microbial Isolation, Identification, and Effects on Mycelial Growth

In total, 81 bacterial and 15 fungal strains were isolated from *P. umbellatus* sclerotia. We then performed a co-culture experiment with *P. umbellatus* mycelia and isolates. The results are presented in Tables S5–S8. Among these bacterial isolates, 70 inhibited mycelial growth, whereas 8 had no effect. However, only three bacterial isolates enhanced the growth of mycelium, and the mycelial diameter with these isolates was 5.88 (FN2), 5.58 (FL19), and 5.87 cm (CL15). (Table S7).

Using EzBioCloud, FN2 was identified as *Acinetobacter* sp., FL19 was *Raoultella* sp., and CL15 was *Pseudomonas* sp. (Table S9). Among the fungal isolates, 12 inhibited mycelial growth, whereas 3 had no effect (Table S8). Therefore, we hypothesized that the growth of *P. umbellatus* was directly associated with bacterial assistance.

### 3.4. Plant Growth-Promoting (PGP) Traits of Bacterial Isolates

To further characterize the mycelial growth promotion by the three bacterial isolates (FN2, FL19, and CL15), we investigated their PGP traits (Figure S1). All three isolates produced IAA with PS and siderophore production activities, and their IAA-producing capacity was in the range of 5.6–73.7 μg/mL. The PS activity of the isolates was assessed by measuring the size of their transparent halos. The results indicated that FN2 exhibited the highest PS ability, with a D/d value of 4.8. An orange halo was observed around the colonies of the three isolates on CAS agar, suggesting the production of siderophores. The D/d value reached 2.2 in FN2 (Table S10). Furthermore, nitrogen fixation of the three isolates was qualitatively examined, and isolates FL19 and CL15 grew normally on an Ashby nitrogen-free medium.

### 3.5. Promotion of P. umbellatus Mycelial Growth by Multiple Isolates

The antagonism test results indicated no antagonistic interactions between FN2, FL19, and CL15. We co-cultured multiple isolates with four groups (FN2-CL15, FN2-FL19, CL15-FL19, and FN2-CL15-FL19) based on combinations of 2–3 single isolates (Table S11). Moreover, we co-cultured *P. umbellatus* mycelia with single or multiple isolates of bacteria for 20 days.

**Figure 3:**
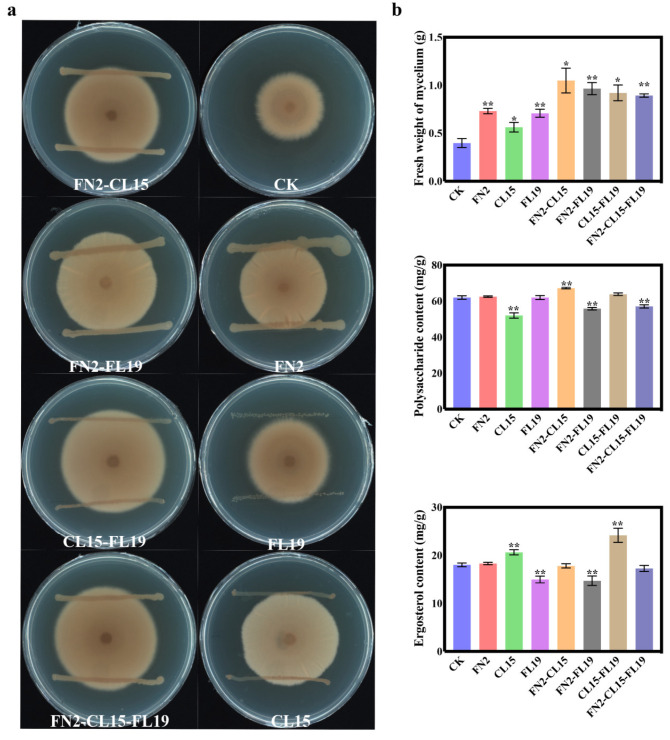
Growth-promoting effects of the three isolates. (**a**) Co-culture of bacterial fermentation broth and *Polyporus umbellatus*. (**b**) Bacterial isolates and their compound formulation increase the mycelium diameter as well as the ergosterol and polysaccharide contents of *P. umbellatus*. CK, *P. umbellatus* mycelium without bacterial fermentation solution. The data are presented as means ± SEM. *, compared with CK, *p* < 0.05; **, *p* < 0.01.

We subsequently determined the fresh weight and the ergosterol and PUP contents of *P. umbellatus* mycelia. All groups exhibited increased mycelial fresh weight, with multiple isolate treatment groups substantially increasing the fresh weight compared to single isolate treatment groups. Notably, the mixed bacterial inoculation of FN2-CL15 significantly increased the fresh weight of mycelia, which was 2.40-fold higher than that of the control group (Figure 3b). In the CL15 and FL19-CL15 groups, the ergosterol content of the *P. umbellatus* mycelium was significantly increased (20.64 and 24.19 mg/g, respectively) compared to that in the control group (18.03 mg/g) (Figure 3b). However, only the FN2-CL15 group showed an increase in the PUPS content of the *P. umbellatus* mycelia, which was 1.08-fold higher than that of the control group.

## 4. Discussion

Fungal growth and development can be divided into vegetative and reproductive stages. Temperature, moisture, and CO_2_ are factors that can impact the growth and development of edible fungi through several metabolic processes. Biological factors can also have a significant influence on the development cycle of edible mushrooms, in addition to the growing conditions. Despite increasing comprehension of the microbiome within the soil or mycorrhizosphere of edible fungal habitats, such as *Morchella sextelata*, *Stropharia rugosoannulata*, *Tuber melanosporum*, and *Agaricus bisporus*, investigations regarding the microecology of *P. umbellatus* sclerotia production remain scarce [25,26,27,28].

In the Qinling Mountains of China, we observed that *P. umbellatus* sclerotia are predominantly cultivated in forests, where mature *P. umbellatus* sclerotia are diced and co-cultured with *Armillaria* spp. The growth of *P. umbellatus* sclerotia primarily relies on *Armillaria* spp. for essential nutrients. Diced *P. umbellatus* sclerotia require at least two years to develop into mature *P. umbellatus* sclerotia; consequently, such a long cultivation cycle impairs the production of *P. umbellatus*. Previous research conducted in our laboratory has demonstrated that the mycorrhizosphere bacterium *Rahnella sp*. HPDA25 enhances the growth of both *Armillaria gallica* and its parasitic host, *Gastrodia elata* [6]. Thus, we hypothesized that a similar mechanism exists for the growth of *P. umbellatus*. In the present study, we found that three bacterial strains, FN2, FL19, and CL15, promoted the growth of *P. umbellatus* mycelia. We performed a BLAST analysis on the sequences of these three isolates and compared them with the OTU results. This analysis revealed that FN2 corresponds to OTU 42 with 99.07% similarity, and CL15 corresponds to OTU 136 with 100% similarity (Table S12). FN2 (OTU 42, *Acinetobacter*) and CL15 (OTU 136, *Pseudomonas*) exhibited higher relative abundances in WBP than in FBP (0.68% and 0.13%, respectively).

Edible mushrooms serve as hosts for a wide range of beneficial bacteria. Various beneficial species, including *Pseudomonas* and *Bacillus*, notably enhance the overall fresh matter production of mushrooms and expedite the harvesting process [29]. Members of the genera *Acinetobacter* and *Pseudomonas* are IAA producers in the rhizosphere, capable of solubilizing phosphates and producing siderophores [30,31]. *Acinetobacter* can perform phenolic acid degradation, aiding in continuous crop disturbances [32,33]. Recently, there has been an increasing interest in lactic acid bacteria as a potentially beneficial class of bacteria for combating phytopathogens [34]. Our study also confirms this, as both FN2 and CL15 produced IAA. Furthermore, FN2 plays a role in solubilizing phosphates and producing siderophores, and CL15 exhibits nitrogen fixation ability. Nitrogen and phosphorus levels and soil fertility positively influence fungus growth. *Acinetobacter* and *Pseudomonas* belong to the phylum Proteobacteria, and their frequent co-occurring interactions with bacteria may contribute to the high yield of *P. umbellatus.* In a previous study, a mixture of three isolates (*Enterobacter cloacae*, *Pseudomonas azotoformans*, and *Bacillus subtilis*) enhanced the biomass and size of *Jerusalem artichoke* tubers of host plants [35]. Moreover, the simultaneous fermentation of *Bacillus subtilis* and *Trichoderma harzianum* enhances plant growth, whereas the co-fermentation of *Rhizopus nigricans* and *Trichoderma pseudokoningii* synergistically controls *Fusarium oxysporum* [36].

Fungi and beneficial bacteria have a widespread connection that promotes fungal development. While the relationship between mushrooms and their surroundings has not been extensively studied, microorganisms play a vital role in different phases of mushroom production. To perform a comprehensive analysis of the beneficial bacteria present in the growing media of non-casing soil *P. umbellatus*, investigating their compositions and functions, examining their interactions with *P. umbellatus*, and devising strategies to enhance production efficiency and quality are essential. Moreover, mushroom production could be improved by gaining a deeper understanding of the composition, function, and changes within the mushroom microbiome to create bioinoculant supplements for mushroom cultivation [37].

Our study demonstrates that a mixture of two isolates (*Acinetobacter* sp. FN2 and *Lactococcus* sp. CL15) stimulated the growth and PUP content of *P. umbellatus* mycelia. Bacteria that interact with one another can be utilized as agronomic amendments to enhance mushroom productivity by stimulating growth. The present study provides initial evidence of the diverse array of interacting bacteria involved in the growth stages of *P. umbellatus*, ultimately facilitating the efficient synthesis of PUPs. To the best of our knowledge, this is the first study to investigate the microbial community structure of *P. umbellatus* sclerotium. This study reports new bioactive microbial resources that can be used to develop fertilizers or pesticides to further enhance the growth and PUP content of *P. umbellatus* mycelia. However, as this study was limited to the laboratory level, there is a need for longer-term studies focused on the impact of artificial bacterial communities on PUP accumulation under field conditions.

## 5. Conclusions

Our study represents the first investigation into the growth-promoting and PUP-promoting activities of identified bacterial strains and their chemical formulations. Further research is necessary to understand the influence of companion bacterial chemical formulations on *P. umbellatus* sclerotium formation. Nonetheless, our study offers valuable insights into harnessing resources from sclerotium-producing strains. We believe that incorporating companion bacteria has the potential to substantially enhance the generation of sclerotia and PUPs in large-scale cultures of *P. umbellatus*.

## Data Availability

The stated raw sequence data are maintained in the Genome Sequence Archive at the National Genomics Data Center, China National Center for Bioinformation/Beijing Institute of Genomics, Chinese Academy of Sciences (GSA: CRA014771), which may be viewed at https://ngdc.cncb.ac.cn/gsa (accessed on 25 January 2024.). The 16S rRNA gene sequences of strain FN2, FL19, and CL15 are uploaded in GenBank with the accession numbers PP204009–PP204011.

## Supplementary Materials

The following supporting information can be downloaded at https://www.mdpi.com/article/10.3390/jof10060386/s1. Figure S1: Plant growth-promoting (PGP) traits of the bacterial isolates; Table S1: Top 10 phylum in terms of relative abundance of bacteria; Table S2: Top 30 genera in terms of relative abundance of bacteria; Table S3: Top 10 phylum in terms of relative abundance of fungi; Table S4: Top 30 genera in terms of relative abundance of fungi; Tables S5–S7: Mycelial diameter of P. umbellatus after co-culture with bacteria; Table S8: Co-culture of fungal strains with P. umbellatus mycelium; Table S9: Identification of the three potent isolates using EzBioCloud; Table S10: PGP traits of the bacterial isolates; Table S11: Antagonism test results; Table S12: Relative abundance of the three isolates.
