# Supplementary material for: Identification of Growth-Promoting Bacterial Resources by Investigating the Microbial Community Composition of Polyporus umbellatus Sclerotia
Source: J Fungi (Basel). 2024 May 27;10(6):386. doi: 10.3390/jof10060386 (PMC11205113; doi:10.3390/jof10060386)
Supplement: Supplementary file 1 [file jof-10-00386-s001.zip › jof-2994823-supplementary.pdf]

Supplementary Material

Table S1. Top 10 phylum in terms of relative abundance of bacteria (%).

Taxonomy	WBP (%)	UWB (%)	FBP (%)	UFB (%)
Proteobacteria	37.56	35.77	34.28	32.66
Unidentified bacteria	15.64	18.30	14.99	20.60
Acidobacteriota	11.82	16.38	6.29	16.25
Bacteroidota	4.77	4.18	9.09	5.42
Actinobacteria	7.30	5.27	4.75	3.83
Verrucomicrobiota	3.37	2.84	3.30	3.49
Actinobacteriota	4.57	2.62	1.34	1.64
Firmicutes	0.40	0.79	1.40	0.86
Chloroflexi	1.86	1.67	1.61	1.98
Myxococcota	1.01	1.32	1.48	1.49
Others	11.68	10.84	11.44	11.77

Table S2. Top 30 genera in terms of relative abundance of bacteria (%).

Taxonomy	WBP (%)	FBP (%)
Serratia	0.04	3.75
Massilia	4.07	0.81
Pseudomonas	0.57	1.76
Flavobacterium	0.58	0.43
Sphingomonas	4.76	5.37
Bradyrhizobium	5.62	1.40
Burkholderia-Caballeronia-Paraburkholderia	2.23	1.53
Pedobacter	0.23	1.48
Candidatus-Solibacter	1.49	2.29
Bryobacter	1.76	3.43
Dyella	0.73	0.12
Candidatus-Udaeobacter	1.67	1.00
Acinetobacter	0.00	0.32
Granulicella	0.87	0.46
Acidothermus	1.55	0.08
Escherichia-Shigella	0.11	0.04
Bacteroides	0.11	0.01
Pantoea	0.13	0.17
Chryseobacterium	0.08	0.58
Puia	0.71	1.11
Novosphingobium	0.26	0.37
Unidentified-*Alphaproteobacteria*	0.73	0.30
Luteibacter	0.27	0.09
Ellin6067	0.30	0.46
Halomonas	0.00	0.56
Paenarthrobacter	0.25	0.43

Acidibacter	0.98	0.43
Edaphobaculum	0.65	0.99
RB41	0.36	0.58
Sphingobacterium	0.00	0.25
Others	68.82	69.38

Table S3. Top 10 phylum in terms of relative abundance of fungi (%).

Taxonomy	WBP (%)	UWB (%)	FBP (%)	UFB (%)
Ascomycota	53.24	23.57	41.95	50.11
Basidiomycota	19.83	37.49	6.68	2.41
Mortierellomycota	12.50	23.63	15.14	10.11
Rozellomycota	5.13	2.19	2.93	0.90
Glomeromycota	0.19	0.12	4.33	0.97
Monoblepharomycota	0.04	0.06	0.02	0.13
Chytridiomycota	0.24	0.37	0.68	1.22
Mucoromycota	0.16	0.28	0.43	0.17
Aphelidiomycota	0.02	0.01	0.07	0.04
Olpidiomycota	0.03	0.03	0.00	0.00
Others	8.63	12.23	27.75	33.93

Table S4. Top 30 genera in terms of relative abundance of fungi (%).

Taxonomy	WBP (%)	FBP (%)
Trichoderma	11.26	9.90
Sarocladium	0.01	0.05
Hygrophorus	6.09	0.00
Mortierella	11.66	14.65
Amanita	0.10	0.02
Fusarium	1.30	4.8
Laccaria	0.58	0.01
Neohelicomyces	2.22	2.75
Inocybe	2.42	0.03
Graphilbum	0.083	4.01
Leptodontidium	5.31	3.29
Xerocomus	0.29	0.00
Vanrija	0.04	2.83
Ascocoryne	0.55	0.00
Gigaspora	0.03	3.68
Mariannaea	3.20	1.21
Boletus	0.16	0.00
Penicillium	0.42	0.64
Octaviania	0.48	0.01
Metarhizium	1.36	0.29
Others	34.84	46.47

Table S5. Mycelial diameter *P. umbellatus* after co-culture with bacteria (Mean ± SD).

Isolates	Diameter of mycelium (cm)
CK	6.90 ± 0.77
FN1	0.97 ± 0.30***
FN3	6.62 ± 0.57
FN4	6.85 ± 1.23
FN5	3.15 ± 1.17***
FL6	3.78 ± 0.66***
FL7	0.60 ± 0.00***
FT9	1.42 ± 0.33***
FT10	4.93 ± 1.40*
FL11	2.53 ± 0.60***
FY12	2.95 ± 0.20***
FL13	2.33 ± 0.23***
FR14	2.82 ± 0.41***
FT15	1.33 ± 0.07***

Compared with the CK group, *, $P < 0.05$; ***, $P < 0.001$.

Table S6. Mycelial diameter of *P. umbellatus* after co-culture with bacteria (Mean ± SD).

Isolates	Diameter of mycelium (cm)
CK	5.67 ± 0.40
WT1	2.58 ± 0.46***
WT2	2.35 ± 0.46***
WT3	2.13 ± 0.65***
WT4	3.40 ± 0.23***
WT（R）6	2.42 ± 0.41***
WT7	2.85 ± 0.40***
WT8	4.37 ± 0.38***
WT9	2.32 ± 0.66***
WT12	5.25 ± 0.30
WT13	0.80 ± 0.06***
WR15	4.93 ± 0.30**
WN16	2.50 ± 0.43***
WN17	4.77 ± 0.57*
WR18	3.02 ± 0.63***
WY19	4.18 ± 0.89**
WT20	2.42 ± 0.30***
WT21	2.55 ± 0.41***
WT22	3.48 ± 0.28***
WR23	3.95 ± 0.11***
WL24	3.68 ± 0.12***
CL1	2.93 ± 0.75***
CL2	1.90 ± 0.28***
CR3	2.75 ± 0.54***

CL4	3.82 ± 0.87**
CL5	1.60 ± 0.22***
CL6	0.60 ± 0.00***
CL7	0.60 ± 0.00***
CL8	4.65 ± 0.63*
CR9	0.60 ± 0.00***
CR10	4.85 ± 0.31**
CL11	0.60 ± 0.00***
CT12	0.60 ± 0.00***
CL13	5.12 ± 0.40
CL(Y)14	0.85 ± 0.08***
CY16	1.82 ± 0.49***
CR17	4.90 ± 0.29**
CN18	4.78 ± 0.54*
CR19	5.24 ± 0.66
CL20	4.85 ± 0.55*
CL21	0.60 ± 0.00***
CY22	0.60 ± 0.00***
CY23	0.60 ± 0.00***
CY24	0.60 ± 0.00***
CY25	0.60 ± 0.00***
CL26	0.60 ± 0.00***
CT27	4.90 ± 0.90
CR28	0.60 ± 0.00***
CT29	5.13 ± 0.38
CT30	3.82 ± 0.07***
CT31	3.70 ± 0.29***
CT32	1.55 ± 0.42***

CT(R)33	$1.63 \pm 0.05^{***}$
CL34	5.25 ± 0.69
CN35	$0.60 \pm 0.00^{***}$
CR36	$0.60 \pm 0.00^{***}$

Compared with the CK group, *, $P < 0.05$; **, $P < 0.01$; ***, $P < 0.001$.

Table S7. Mycelial diameter of *P. umbellatus* after co-culture with bacteria (Mean ± SD).

Isolates	Diameter of mycelium (cm)
CK	4.65 ± 0.43
FN2	$5.88 \pm 0.25^{***}$
FY8	$0.60 \pm 0.00^{***}$
FN16	$0.60 \pm 0.00^{***}$
FN17	$0.60 \pm 0.00^{***}$
FR18	$0.60 \pm 0.00^{***}$
FL19	$5.58 \pm 0.55^{*}$
FL20	$3.54 \pm 0.77^{*}$
FL21	3.90 ± 1.35
WL5	$3.87 \pm 0.44^{*}$
WL10	$0.60 \pm 0.00^{***}$
WL11	$2.72 \pm 0.68^{***}$
WL14	$0.60 \pm 0.00^{***}$
CL15	$5.87 \pm 0.56^{**}$

Compared with the CK group, *, $P < 0.05$; **, $P < 0.01$; ***, $P < 0.001$.

Table S8. Co-culture of fungal strains with *P. umbellatus* mycelium (Mean ± SD).

Isolates	Diameter of mycelium (cm)
CK	4.36 ± 0.26
ZM1	2.40 ± 0.13***
ZM2	3.95 ± 0.37
ZM3	3.40 ± 0.46**
ZM4	3.37 ± 0.60**
ZM5	0.73 ± 0.15***
ZM6	3.65 ± 0.31**
ZM7	0.63 ± 0.05***
ZM8	2.45 ± 0.32***
ZM9	2.47 ± 0.29***
ZM10	2.52 ± 0.71***
ZM11	0.75 ± 0.09***
ZM12	2.35 ± 0.17***
ZM13	0.62 ± 0.04***
ZM14	4.15 ± 0.66
ZM15	4.07 ± 0.60

Compared with the CK group, **, $P < 0.01$; ***, $P < 0.001$.

Table S9. Identification of the three potent isolates using EzBioCloud.

Isolates	Top-hit taxon	Similarity (%)	Completeness (%)
FN2	*Acinetobacter JFYA_s*	99.78	92.50
CL15	*Pseudomonas MDEN_s*	99.68	85.50
FL19	*Raoultella ornithinolytica*	99.35	92.50

Table S10. PGP traits of the bacterial isolates (Mean ± SD).

Isolates	IAA content (μg/mL)	PS activity (D/d)	Siderophore production (D/d)
FN2	11.71 ± 0.29^{b}	4.80 ± 0.42^{a}	2.2 ± 0.12^{a}
FL19	42.36 ± 0.53^{a}	1.10 ± 0.03^{bc}	1.5 ± 0.00^{c}
CL15	3.93 ± 0.30^{c}	1.60 ± 0.09^{b}	1.7 ± 0.05^{b}

Different letters indicate $P < 0.05$.

Table S11. Antagonism test results.

Isolates	FL19	CL15
FN2	+	+
FL19		+

+ denoted that the strains could be cocultured with each other.

Table S12. Relative abundance of the three isolates (%).

Isolates	OTUs	Similarity (%)	Relative abundance			
			WBP	FBP	UWB	UFB
FN2	OTU 42	99.07	0.68	0.12	0.02	0.01
CL15	OTU 136	100	0.13	0.38	0.00	0.00
FL19	OTU 347	97.67	0.01	0.01	0.01	0.00

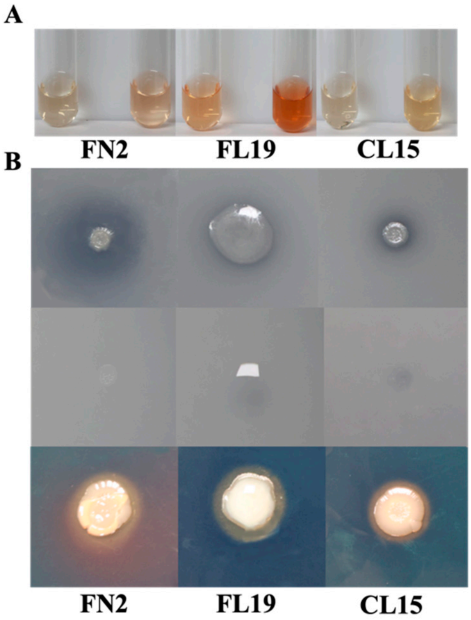

Figure S1. Plant growth-promoting (PGP) traits of the bacterial isolates. A: IAA production. B: Phosphate solubilization, nitrogen fixation, and siderophore production.
